# Molecular basis of natural variation and environmental control of trichome patterning

**DOI:** 10.3389/fpls.2014.00320

**Published:** 2014-07-03

**Authors:** Marie-Theres Hauser

**Affiliations:** Department of Applied Genetics and Cell Biology, University of Natural Resources and Life SciencesVienna, Austria

**Keywords:** trichome development, natural variation, QTL, GWAS, abiotic, biotic, defense

## Abstract

Trichomes are differentiated epidermal cells on above ground organs of nearly all land plants. They play important protective roles as structural defenses upon biotic attacks such as herbivory, oviposition and fungal infections, and against abiotic stressors such as drought, heat, freezing, excess of light, and UV radiation. The pattern and density of trichomes is highly variable within natural population suggesting tradeoffs between traits positively affecting fitness such as resistance and the costs of trichome production. The spatial distribution of trichomes is regulated through a combination of endogenous developmental programs and external signals. This review summarizes the current understanding on the molecular basis of the natural variation and the role of phytohormones and environmental stimuli on trichome patterning.

## INTRODUCTION

Plants have evolved sophisticated morphological and chemical systems to cope with biotic and abiotic challenges. Differentiated epidermal cells such as leave hairs or trichomes represent one of these systems. Trichomes develop on above ground organs including seeds and fruits and occur in a large variety of morphologies. They can be single-celled or multicellular, branched or unbranched, and glandular or non-glandular, characteristics often used for species identification (Luckwill, 1943; Beilstein et al., 2006, 2008). Trichomes have a range of protective functions however producing them is costly, depend on plant resource availability and can have negative impacts on plant growth and vigor (Wilkens et al., 1996). Therefore trichome production often underlies qualitative and quantitative variation in most plant species.

## TRICHOMES AND ABIOTIC FACTORS

In adverse environments, trichomes are beneficial because they influence the water balance, protect photosynthesis and reduce photoinhibition. Their density is negatively correlated with the rate of transpiration (Choinski and Wise, 1999; Benz and Martin, 2006) and that of carbon dioxide diffusion (Ehleringer et al., 1976; Galmés et al., 2007). The pubescent nature of plants growing in extreme alpine and Mediterranean habitats correlates with trichome’s ability to protect the underlying tissue from sunlight by increasing the reflectance and reducing the heat load. Many plants accumulate also UV-absorbing compounds such as flavonols in trichomes which further protect the underlying photosynthetic tissues from damaging amount of UV-A and UV-B radiations (Karabourniotis et al., 1995, 1998; Karabourniotis and Bornman, 1999; Tattini et al., 2000, 2007; Morales et al., 2002; Liakopoulos et al., 2006; Yan et al., 2012). Evidence that trichomes are structural adaptations to low temperature and enhance tolerance to freezing came from studies on birch where frost increased rapidly the density of glandular trichomes (Prozherina et al., 2003). Some heavy metal tolerant plants accumulate heavy metals in trichomes serving for detoxification purposes (Choi et al., 2001, 2004; Marmiroli et al., 2004; Freeman et al., 2006; Harada and Choi, 2008; Sarret et al., 2009; Quinn et al., 2010). Heavy metal loaded trichomes might contribute to elemental defense strategies (Boyd, 2012; Cheruiyot et al., 2013; Kazemi-Dinan et al., 2014).

## TRICHOMES AND BIOTIC CHALLENGES

Many studies show that trichomes serve as physical barrier against biotic stressors such as insects, herbivores, fungal infections, and even parasitic plants (Peiffer et al., 2009; Runyon et al., 2010; Tian et al., 2012). Solanaceous species such as tomato and related species produce a variety of trichomes. The long multicellular type I trichomes that have a small glandular vesicle at the tip on hypocotyls for example effectively hinder the infection of tomato (*Solanum lycopersicum*) with its parasite *Cuscuta pentagona* (Runyon et al., 2010). Recent studies in several tomato wild relatives found that the presence, density, longevity and size of type I and the shorter multicellular type IV glandular trichomes correlates with resistance against whitefly (Firdaus et al., 2012, 2013). Oviposition and feeding experiments with the specialist moth *Plutella xylostella* on different *Arabidopsis thaliana* accessions showed that oviposition varied significantly among populations and could partly be explained by a negative relationship between trichome density and egg number, and a positive relationship between plant size and egg number (Handley et al., 2005). Experiment with glabrous and hairy *Arabidopsis lyrata* morphs and larvae of *Plutella xylostella* show that trichomes increased resistance to leaf damage and reduced oviposition in adult plants (Sletvold et al., 2010). However, in young plants that develop fewer trichomes this effect was not significant (Puentes and Ågren, 2013). The larvae of the crucifer-feeding beetle, *Phaedon brassicae,* grew slower on hairy leaves of *Arabidopsis halleri*. Hairy leaves were less damaged when glabrous leaves were abundant in free choice experiments (Sato et al., 2014).

While non-glandular trichomes can be seen as structural defenses, glandular trichomes are also a source of highly interesting biomolecules (Shepherd et al., 2005; Liu et al., 2006; Kang et al., 2010). Apart from the above mentioned flavonols, glandular trichomes synthesize and/or store other highly valuable secondary metabolites such as terpenoids, phenylpropenes, methyl ketones (Fridman et al., 2005; Ben-Israel et al., 2009), acyl sugars (Schilmiller et al., 2012; Stout et al., 2012; Xu et al., 2013), and proteinase inhibitors (Tian et al., 2012) and thus contribute to the chemical repertoire of defense strategies. Given that trichomes provide both structural and chemical defense systems against herbivores and pathogens they are appealing targets for breeding (Gruber et al., 2006; Glas et al., 2012).

Controversial is the effect of trichomes for fungal infections: While damaged trichomes are often the starting point for colonialization with powdery mildew (*Erysiphe necator*) on grapevine buds (Rumbolz and Gubler, 2005), *Botrytis cinerea* on harvested tomato (Charles et al., 2008), *Phoma clematidina* on clematis (Van De Graaf et al., 2002) or *Beauveria bassiana* on poppy (Landa et al., 2013), glandular trichomes are often able to secrete exudates with antifungal activity as shown in a wild potato species (*Solanum berthaultii*) and its resistance to *Phytophthora infestans* (Lai et al., 2000). The disease incidence correlated negatively with the density and polyphenol-oxidase activity of short type A trichomes that have a four-lobed membrane-bound gland at their tips (Lai et al., 2000). In the infection of chickpea (*Cicer arietinum*) with *Ascochyta rabiei* the concentration of a highly acidic trichome exudate is crucial. At low concentrations the exudate promotes germination of *Ascochyta rabiei* conidia while at high concentrations germination is inhibited (Armstrong-Cho and Gossen, 2005). Also glandular trichomes of tobacco (*Nicotiana tabacum*) produce a potent inhibitor, T-phylloplanin, which inhibits germination of the oomycete *Peronospora tabacina* (Kroumova et al., 2007). The effect of trichomes is specific for the resistance to fungi. For example, while in *Arabidopsis thaliana* the infection with the soil-borne pathogen *Rhizoctonia solani* is not affected by trichome density, *gl1* mutants were more resistant and the *try* mutant with clustered trichomes had an enhance colonialization with *Botrytis cinerea* (Calo et al., 2006). However, *Arabidopsis thaliana* transgenes expressing the antifungal α-1,3-glucanase of *Trichoderma harzianum* in trichomes were more resistant to *Botrytis cinerea* demonstrating that trichomes can be engineered to increase resistance to fungal pathogens (Calo et al., 2006).

## REGULATION OF TRICHOME DENSITY IN *Arabidopsis* AND OTHER BRASSICACEAE

Classical molecular genetic approaches of the model plant *Arabidopsis thaliana* identified major regulators of trichome development on leaves, stems and petiols. They fall into two classes: positive (mutants develop less trichomes) and negative regulators (mutants develop more and/or clusters of trichomes; for reviews see Balkunde et al., 2010). The positive regulators belong to three protein classes: a WD40 protein TRANSPARENT TESTA GLABRA1 (TGG1; Galway et al., 1994; Walker et al., 1999), three R2R3 MYB-related transcription factors GLABRA1 (GL1, MYB23, MYB5; Oppenheimer et al., 1991; Kirik et al., 2005; Song et al., 2009; Tominaga-Wada et al., 2012) and four basic helix-loop-helix (bHLH)-like transcription factors GLABRA3 (GL3; Payne et al., 2000), ENHANCER OF GLABRA3 (EGL3), TRANSPARENT TESTA (TT8; Zhang et al., 2003), and MYC-1 (Zhao et al., 2012). They act partially redundantly and form a multimeric activator complex, also known as MYB-bHLH-WD40 (MBW) complex which binds the promoter of *GLABRA2 (GL2). GL2* encodes a homeodomain protein required for subsequent phases of trichome morphogenesis such as endoreduplication, branching, and maturation of the cell wall. The negative regulators are seven partially redundant single-repeat MYBs such as CAPRICE (CPC), TRIPTYCHON (TRY), ENHANCER OF TRY AND CPC 1, 2, 3 (ETC1, ETC2, ETC3), and TRICHOMELESS1 and 2 (TCL1, TCL2; Wester et al., 2009; Edgar et al., 2014; Wang and Chen, 2014). For most of them it has been shown that they act in a non-cell-autonomous manner. The single-repeat MYBs lack the C-terminal activation domain and inhibit the activator complex by replacing the R2R3 MYB-related transcription factors and thereby suppress trichome initiation in adjacent cells. While some of the positive and negative regulators are specific for trichome patterning others are also involved in root hair development, anthocyanin biosynthesis (Nemie-Feyissa et al., 2014), and seed coat mucilage production (Zhang et al., 2003; Song et al., 2009).

## HETEROBLASTY AND HORMONAL CONTROL OF TRICHOME DENSITY

Trichome density is developmentally regulated. For example, *Arabidopsis* rosette leaves have trichomes only on the adaxial side and the number increases with the age of plants so that early leaves develop fewer and later more trichomes. On the other hand cauline leaves develop mainly abaxial and lack adaxial trichomes. This heteroblasty varies in different accessions (Larkin et al., 1996; Telfer et al., 1997; Gan et al., 2006; Hilscher et al., 2009) and is influenced by the photoperiod (Chien and Sussex, 1996).

Moreover hormones such as gibberellin (GA) promote trichome initiation and morphogenesis (Telfer et al., 1997; Perazza et al., 1998; Gan et al., 2006) by inducing *GL1* expression. The original observation was that the GA biosynthesis mutant, *ga1-3,* develops less adaxial trichomes on leaves (Chien and Sussex, 1996) and application of GA restored and induces trichome production. Furthermore GA regulates also later stages in trichome development since mutants of the SPINDLY repressor of GA signaling not only develop glabrous sepals but also over-branched leaf trichomes (Perazza et al., 1998; Silverstone et al., 2007). For the effect of GA on trichome initiation on inflorescence organs four redundantly acting C2H2 transcription factors have been identified: GLABROUS INFLORESCENCE STEMS (GIS, GIS2), ZINC FINGER PROTEIN 8 and 5 (ZFP8, ZFP5). They act upstream of GL1 and are involved in the action of cytokinin on trichome initiation (Gan et al., 2006, 2007a,b; Zhou et al., 2013).

As mentioned above trichomes can be induced by wounding and insect attack (Larkin et al., 1996; Yoshida et al., 2009) and the plant hormones involved in signaling these stresses are jasmonic acid (JA) and salicylic acid (Traw and Bergelson, 2003). Recently is has been shown that the JA receptor, CORONATINE-INSENSITVE1 (COI1), is involved in JA induced trichome production in tomato and *Arabidopsis* (Li et al., 2004; Qi et al., 2011) and that several repressors of JA signaling, JAZ1, 2, 5, 6, 8, 9, 10, 11 are able to interact with components of the activator complex such as EGL3, GL3, TT8, MYB75, GL1 (Qi et al., 2011).

The positive effect of JA on trichome production is antagonized by salicylic acid. Reduced trichome development was observed after salicylic acid treatment or on mutants with elevated salicylic acid levels such as the *CONSTITUTIVE EXPRESSION OF PR GENE* (*cpr)* mutants (Bowling et al., 1997; Traw and Bergelson, 2003; An et al., 2011).

## NATURAL VARIATIONS AS SOURCE OF NOVEL TRICHOME REGULATORS

**FIGURE 1:**
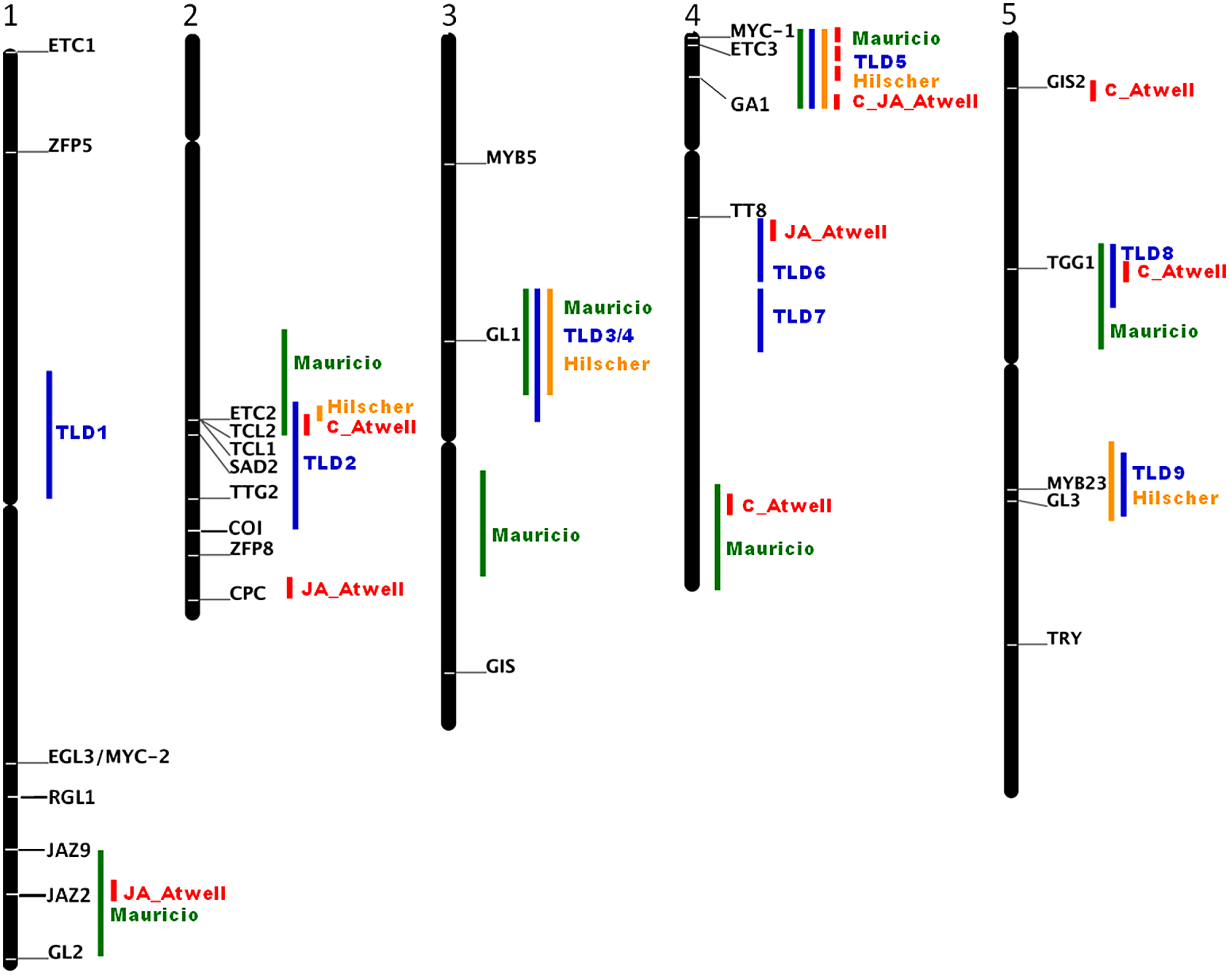
**Schematic representation of the loci influencing trichome density and number on rosette leaves from QTL analyses with different accessions (green; Mauricio, 2005), (blue; Symonds et al., 2005), (orange; Hilscher et al., 2009), and GWAS (red; Atwell et al., 2010).** Indicated are the candidate genes that have been shown to influence trichome pattering and as bars the position of the QTL and GWAS loci. For GWAS only regions are indicated that contain the 200 most significant SNPs. AT3G27920 *GL1*; AT5G40330 *MYB23*; AT3G13540 *MYB5*; At5g41315 *GL3*; At1g63650 *EGL3/MYC-2*; At4g00480 *MYC-1*; At5g24520 *TGG1*; At2g37260 *TTG2*; At1g79840 *GL2*; AT2G30420 *ETC2*; AT2G30424 *TCL2*; AT2G30432 *TCL1*; AT2G46410 *CPC*; AT5G53200 *TRY*; AT1G01380 *ETC1*; AT4G01060 *ETC3*; AT3G58070 *GIS*; AT5G06650 *GIS2*; AT2G41940 *ZFP8*; AT2G31660 *SAD2*; AT1G10480 *ZFP5*; AT4G09820 *TT8*; AT1G66350 RGL1; AT1G70700 JAZ9; AT1G74950 JAZ2; AT2G39940 COI.

Molecular analyses of natural variations of morphological and developmental traits have been a powerful approach to identify new genes important for adaptation to different environments (Assmann, 2013). For example the analyses of natural sequence variations of *GL1* show that in particular the 3′ end is responsible for the glabrous phenotype of the *Arabidopsis thaliana* accession Mir-0, Br-0, Fran-3, PHW-2, 9354, Wil-2, Est as well as for hairless *Arabidopsis lyrata, Arabidopsis halleri, Brassica rapa, Brassica oleracea, Brassica napus,* and radish (*Raphanus sativus)* lines (Hauser et al., 2001; Kärkkäinen and Ågren, 2002; Kawagoe et al., 2011; Li et al., 2011, 2013; Bloomer et al., 2012). Larkin et al. (1993) has experimentally tested the importance of the non-coding 3′ end and postulated that an enhancer downstream of the coding region is essential for the precise expression and function of *GL1* in *Arabidopsis*. However, major phenotypic variations are rarely the effect of only one gene and its natural alleles, more frequently phenotypic variations in natural accessions depend on several partially interacting loci with quite small contributions. Their analysis needs statistical approaches such as quantitative trait locus (QTL) mapping. The first QTL analysis discovered a major locus, named *REDUCED TRICHOME NUMBER (RTN)* in *Arabidopsis thaliana* and used recombinant inbred lines (RIL) derived from a cross between the low trichome density Ler and the medium density Col-accessions (Larkin et al., 1996). This locus was identified in all further QTL analyses with combination of different accessions (Mauricio, 2005; Symonds et al., 2005; Bloomer et al., 2014) and even in a genome wide association study (GWAS; Atwell et al., 2010) as major regulator of trichome density. Hilscher et al. (2009) finally revealed *RTN* as *ETC2* and the K19E amino acid substitution to be responsible for low trichome densities in natural *Arabidopsis thaliana* accessions. However, *ETC2* is not the only gene responsible for trichome density variations. Mauricio (2005) and Symonds et al. (2005) identified each nine QTLs for trichome density in four recombinant inbred mapping populations of *Arabidopsis thaliana.* Most of the identified QTLs regions contain or are in close proximity of known trichome initiation regulators such as *GL2, ETC2, TCL2, TCL1, SENSITIVE TO ABA AND DROUGHT2* (*SAD2*; Gao et al., 2008), *TTG2, CPC, GL1, MYC-1, ETC3, GA1, TT8, GIS, TTG1, MYB23* and *GL3.* For *MYC-1* a non-synonymous substitution was identified in few accession which however did not correlate with trichome density (Symonds et al., 2011). While the sequence variation of *ETC2, TCL2, TCL1,* and *GL1* have been studied the other candidate genes as well as genomic regions without candidate genes such as the loci TLD1 on chromosome 1, TLD6 and TLD7 in the middle of chromosome 3 and others identified with GWAS await closer examinations (**Figure 1**). With the availability of the 1001 *Arabidopsis* genomes association studies of the remaining candidate genes are now straight forward (Ossowski et al., 2008; Cao et al., 2011).

## OUTLOOK AND POTENTIAL OF UNDERSTANDING THE BASIS OF NATURAL VARIATIONS AND ENVIRONMENTAL INFLUENCES ON TRICHOME DENSITY REGULATION

Although the major players of trichome density regulation have been identified in the model plant *Arabidopsis* they are still not sufficient to explain all the naturally occurring variations in this plants species. Great potential for the identification of still missing regulators will come from next generation sequencing possibilities in combination with classical genetic, population genetic and comparative approaches using different plant species. There are already several examples where trichome patterning regulators from wild relatives or even crops and distantly related species such as cotton, tomato and hop have been successfully and functionally studied in the model plant *Arabidopsis* (Wang et al., 2004; Guan et al., 2011; Kocábek and Matoušek, 2013; Tominaga-Wada et al., 2013). However, there are further needs for research determining the molecular basis of the patterning of different types of glandular trichomes and in particular of pharmaceutical and agronomically interesting plant species. Since trichomes serve as morphological, and in cases of glandular trichomes as chemical defense barriers against many abiotic stresses and biotic attacks, increasing their density has great potential to improve broad-spectrum pest and pathogen resistance in crops.

## Conflict of Interest Statement

The author declares that the research was conducted in the absence of any commercial or financial relationships that could be construed as a potential conflict of interest.
